# Energy Conservation via Hydrogen Cycling in the Methanogenic Archaeon Methanosarcina barkeri

**DOI:** 10.1128/mBio.01256-18

**Published:** 2018-07-03

**Authors:** Gargi Kulkarni, Thomas D. Mand, William W. Metcalf

**Affiliations:** a Department of Microbiology, University of Illinois at Urbana-Champaign, Champaign, Illinois, USA; b Carl R. Woese Institute for Genomic Biology, University of Illinois at Urbana-Champaign, Champaign, Illinois, USA; University of California, Irvine

**Keywords:** *Methanosarcina*, energy conservation, hydrogenase, methanogenesis

## Abstract

Energy conservation via hydrogen cycling, which generates proton motive force by intracellular H_2_ production coupled to extracellular consumption, has been controversial since it was first proposed in 1981. It was hypothesized that the methanogenic archaeon Methanosarcina barkeri is capable of energy conservation via H_2_ cycling, based on genetic data that suggest that H_2_ is a preferred, but nonessential, intermediate in the electron transport chain of this organism. Here, we characterize a series of hydrogenase mutants to provide direct evidence of H_2_ cycling. M. barkeri produces H_2_ during growth on methanol, a phenotype that is lost upon mutation of the cytoplasmic hydrogenase encoded by *frhADGB*, although low levels of H_2_, attributable to the Ech hydrogenase, accumulate during stationary phase. In contrast, mutations that conditionally inactivate the extracellular Vht hydrogenase are lethal when expression of the *vhtGACD* operon is repressed. Under these conditions, H_2_ accumulates, with concomitant cessation of methane production and subsequent cell lysis, suggesting that the inability to recapture extracellular H_2_ is responsible for the lethal phenotype. Consistent with this interpretation, double mutants that lack both Vht and Frh are viable. Thus, when intracellular hydrogen production is abrogated, loss of extracellular H_2_ consumption is no longer lethal. The common occurrence of both intracellular and extracellular hydrogenases in anaerobic microorganisms suggests that this unusual mechanism of energy conservation may be widespread in nature.

## IMPORTANCE

ATP is required by all living organisms to facilitate essential endergonic reactions required for growth and maintenance. Although synthesis of ATP by substrate-level phosphorylation is widespread and significant, most ATP is made via the enzyme ATP synthase, which is energized by transmembrane chemiosmotic gradients. Therefore, establishing this gradient across the membrane is of central importance to sustaining life. Experimental validation of H_2_ cycling adds to a short list of mechanisms for generating a transmembrane electrochemical gradient that is likely to be widespread, especially among anaerobic microorganisms.

## INTRODUCTION

An essential requirement for life is the ability to couple exergonic metabolism to the endergonic synthesis of ATP. While some ATP is made by direct phosphorylation of ADP using “high-energy” metabolites such as phosphoenolpyruvate or 1,3-diphosphoglycerate, the vast majority is produced via the enzyme ATP synthase using energy stored in a transmembrane proton (or sodium) gradient. These electrochemical gradients are typically established during the process of electron transport by membrane proteins that couple exergonic redox reactions to generation of an ion motive force by one of three general mechanisms: (i) vectorial proton pumping; (ii) scalar movement of protons across the membrane, as in the Q-cycle or Q-loop; or (iii) coupled reactions that consume protons within the cell and produce protons on the outside (1, 2). Given the importance of this process, it is not surprising that this central aspect of living systems has been the subject of intense study (and at least three Nobel Prizes). Indeed, we now possess a detailed, molecular-level understanding of chemiosmotic energy conservation as it applies to photosynthesis and aerobic respiration in a wide variety of organisms, including eukaryotes, bacteria, and archaea. Nevertheless, unique and sometimes surprising mechanisms for generation of chemiosmotic gradients continue to be found, including sodium-pumping methyltransferases in methanogenic archaea (3), electrogenic formate:oxalate antiporters in bacteria (4, 5), and light-driven, proton-pumping rhodopsins (6).

A controversial, and as yet unproven, mechanism for creating transmembrane proton gradients called H_2_ cycling was proposed by Odom and Peck in 1981 to explain ATP synthesis in sulfate-reducing bacteria (7). In this proposed energy-conserving process, protons in the cytosol are reduced to molecular H_2_ by enzymes known as hydrogenases. The H_2_ so produced then diffuses across the membrane where it is reoxidized by extracellular hydrogenases, releasing protons that contribute to a transmembrane proton gradient that can be used to make ATP. The electrons produced by this reaction are returned to the cytoplasm via a membrane-bound electron transport chain, completing the redox process.

Although H_2_ cycling has been suggested to occur in a number of anaerobic organisms (7–11), the hydrogen cycling hypothesis has not been widely accepted. A key argument against the idea is based on the high diffusion rate of molecular hydrogen. Thus, unless extracellular recapture is exceptionally efficient, hydrogen produced in the cytoplasm would be easily lost, resulting in redox imbalance and presumably cell death. Nevertheless, experimental demonstration of simultaneous production and consumption of H_2_ by Desulfovibrio vulgaris supports the model (12), as does metabolic modeling (13). However, other data are inconsistent with the idea, including the ability of hydrogenase mutants to grow on lactate (14) and the inability of high external H_2_ pressures to inhibit substrate catabolism (15). Thus, the H_2_ cycling model for energy conservation remains unproven.

**FIG 1:**
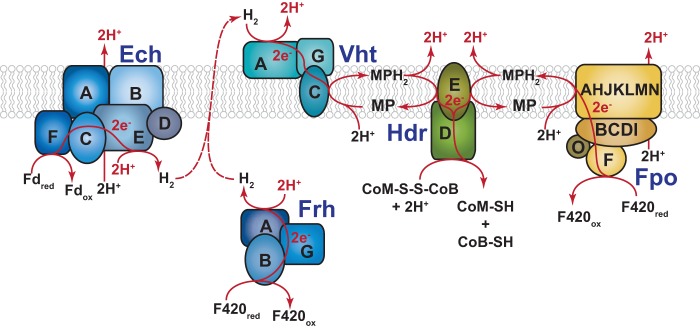
Putative H_2_ cycling electron transport chain of M. barkeri. Growth on C_1_ substrates generates reduced cofactor F420 (F420_red_), which is a hydride carrying cofactor analogous to NADH, and the reduced form of the small electron-carrying protein ferredoxin (Fd_red_). During aceticlastic methanogenesis, only Fd_red_ is produced. These reduced electron carriers are reoxidized in the cytoplasm by the Frh and Ech hydrogenases, respectively, with concomitant consumption of protons to produce molecular H_2_. H_2_ subsequently diffuses out of the cell where it is reoxidized by the Vht hydrogenase, which has an active site located on the outer face of the cell membrane. This reaction releases protons on the outside of the cell and produces reduced methanophenazine (MPH_2_), a membrane-bound electron carrier analogous to ubiquinone. MPH_2_ subsequently delivers electrons to the enzyme heterodisulfide reductase (Hdr), which serves as the terminal step in the *Methanosarcina* electron transport chain. This final reaction regenerates coenzyme B (CoB-SH) and coenzyme M (CoM-SH) from the mixed disulfide (CoM-S-S-CoB), which is produced from the free thiol cofactors during methanogenic metabolism. Electron (e^−^) flow and scalar protons (H^+^) are shown in red. It should be noted that M. barkeri can also reoxidize F420_red_ using the membrane-bound, proton-pumping F420-dehydrogenase (Fpo). Thus, the cell has a branched electron transport chain, and therefore, it is not dependent on H_2_ cycling during growth on methylotrophic substrates (16); however, both pathways for electron transport from F420 have identical levels of energy conservation: namely, 4 H^+^/2e^−^. It should also be noted that the Ech hydrogenase acts as a proton pump in addition to its role in H_2_ cycling, thus electron transport from Fd_red_ during methylotrophic and aceticlastic methanogenesis conserves 6H^+^/2e^−^. Individual subunits of the various enzymes are indicated by capital letters (e.g., A, B, C…).

On the basis of a series of genetic experiments, we proposed that the methanogenic archaeon Methanosarcina barkeri employs H_2_ cycling during growth on one-carbon (C_1_) substrates and acetate (16, 17). During growth on C_1_ compounds such as methanol, the putative cycling pathway would produce H_2_ using the cytoplasmic F420-dependent (Frh) and energy-converting ferredoxin-dependent (Ech) hydrogenases, while H_2_ production during growth on acetate would be mediated solely by Ech. Both pathways would converge on the methanophenazine-dependent hydrogenase (Vht), which is thought to have an active site on the outer face of the cell membrane (18), to consume extracellular H_2_ and deliver electrons to the membrane-bound electron transport chain, where they serve to reduce the coenzyme M-coenzyme B heterodisulfide (CoM-S-S-CoB) produced during the production of methane (Fig. 1). However, these genetic studies remain incomplete because neither the role of Vht nor the production and consumption of hydrogen were examined. Here we explicitly test both, providing strong experimental support for the role of H_2_ cycling in energy conservation in *M. barkeri*.

## RESULTS AND DISCUSSION

### Hydrogenases of M. barkeri.

Three distinct types of hydrogenases are encoded by Methanosarcina barkeri Fusaro (see Fig. S1 in the supplemental material) (19). The F420-reducing hydrogenase (Frh) is a cytoplasmic, three-subunit (α, β, and γ) enzyme encoded by the *frhADGB* operon, which also includes a maturation protease, FrhD (20). This enzyme couples the oxidation/reduction of the deazaflavin cofactor F420 with production/consumption of H_2_. The membrane-bound Vht hydrogenase utilizes the quinone-like electron carrier, methanophenazine, as a cofactor (21). Like Frh, Vht is a three-subunit enzyme encoded by a four-gene operon (*vhtGACD*) that includes a maturation protease, VhtD (19). M. barkeri also contains genes that encode homologs of both the *frh* and *vht* operons (the *freAEGB* and *vhxGAC* operons, respectively); however, multiple lines of evidence suggest that these genes are incapable of producing active hydrogenases (16, 22). Thus, the presence of these genes has no bearing on the results presented herein. The final hydrogenase encoded by M. barkeri is a membrane-bound, energy-converting hydrogenase (Ech), which couples the oxidation/reduction of ferredoxin and H_2_ to the production/consumption of a proton motive force (23, 24). Thus, the enzyme can use proton motive force to drive the endergonic reduction of ferredoxin by H_2_, which is required for CO_2_ reduction during hydrogenotrophic methanogenesis and for biosynthesis during growth by H_2_-dependent reduction of C_1_ compounds (methyl-reducing methanogenesis). During both methylotrophic and aceticlastic methanogenesis, Ech is believed to couple oxidation of reduced ferredoxin to production of proton motive force and H_2_. The hydrogen thus produced would need to be recaptured by Vht in a putative H_2_ cycling process that contributes to proton motive force (Fig. 1) (17).

10.1128/mBio.01256-18.1 FIG S1  Hydrogenase operons in Methanosarcina barkeri. Three distinct types of hydrogenase are encoded by M. barkeri Fusaro. The *frh* and *fre* operons encode putative F420-reducing hydrogenases, while the *vht* and *vhx* operons encode putative methanophenazine-reducing hydrogenases. Genetic and biochemical data show that neither the *fre* nor the *vhx* operon is capable of producing an active hydrogenase under any growth condition yet examined (T. D. Mand, G. Kulkarni, and W. W. Metcalf, 2018 bioRxiv, https://doi.org/10.1101/334656). The *ech* operon encodes a ferredoxin-dependent energy-conserving hydrogenase. The locus tags are shown below each gene, with the prefix “Mbar_” omitted to save space (indicated by an asterisk) in some cases. Download FIG S1, PDF file, 0.7 MB. Copyright © 2018 Kulkarni et al. 2018 Kulkarni et al. This content is distributed under the terms of the Creative Commons Attribution 4.0 International license.

### The cytoplasmic Frh hydrogenase is responsible for production of H_2_ during growth on methanol.

**FIG 2:**
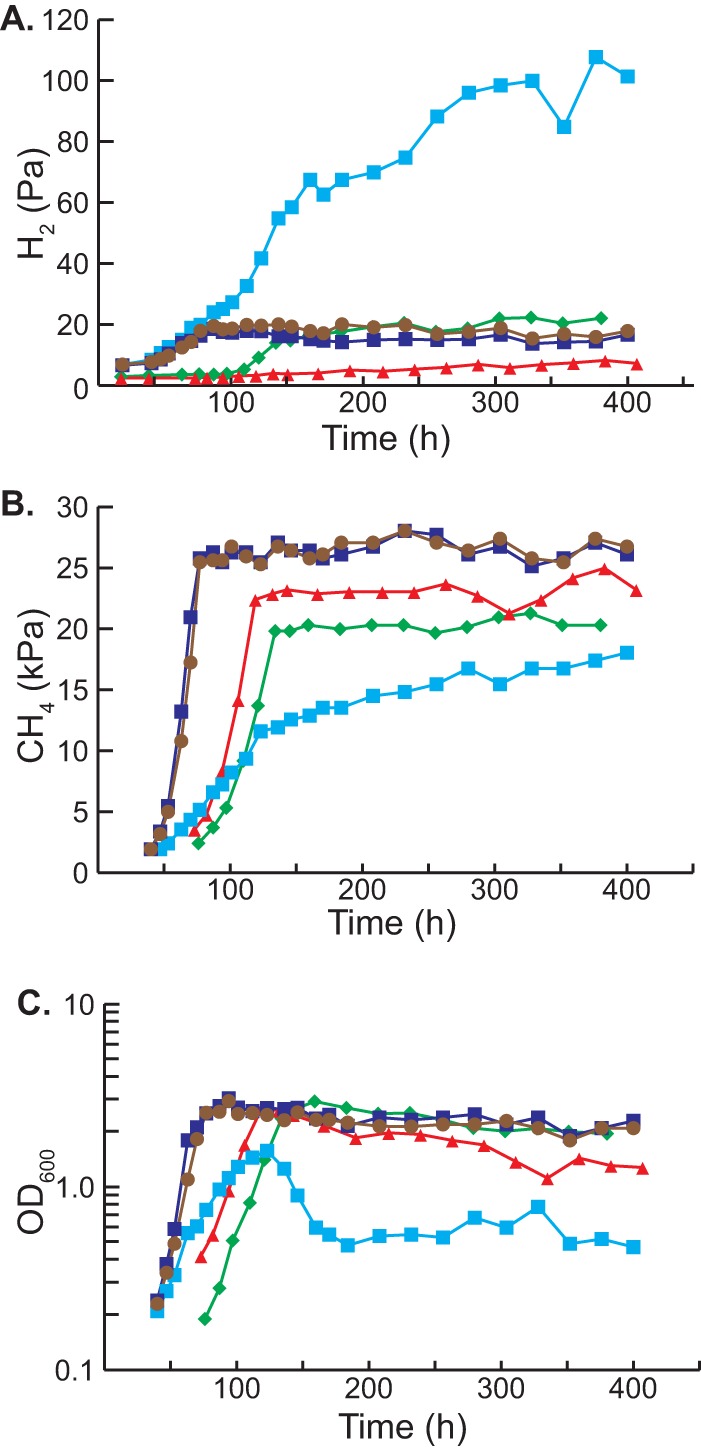
Hydrogen and methane production during methylotrophic growth. (A to C) The partial pressures of H_2_ (A) and methane (B) were monitored during the course of growth (as indicated by optical density [C]) in methanol-containing medium for various M. barkeri strains. Strains used were M. barkeri isogenic parental strain (WWM85 [brown circles]), tetracycline-regulated P_tet_::*vht* mutant (WWM157) with tetracycline (dark blue squares) and without tetracycline (light blue squares), Δ*frh* mutant (WWM115 [red triangles]), and Δ*frh* Δ*vht* double mutant (WWM351 [green diamonds]). Measurements were performed in triplicates as described in Materials and Methods. Complete strain genotypes can be found in Table S1 in the supplemental material.

A number of studies have shown that assorted *Methanosarcina* strains produce H_2_ during growth on methylotrophic and aceticlastic substrates (9, 25–30); however, to our knowledge, this has never been assessed in M. barkeri strain Fusaro. To test this, we quantified the accumulation of CH_4_ and H_2_ during growth on methanol-containing medium (Fig. 2). Consistent with the hydrogen cycling hypothesis, we observed significant H_2_ production, which reached a maximum partial pressure of ca. 20 Pa near the end of exponential growth. As expected, the culture also produced substantial levels of methane. As previously observed (16), a mutant lacking Frh (strain WWM115 [Table S1]) grew at a lower rate than its isogenic parent and produced somewhat smaller amounts of methane. Very little H_2_ (<4 Pa) was produced during growth of the Δ*frh* mutant; however, after growth ceased, the H_2_ concentration slowly rose, reaching a maximum level of 7 Pa. Thus, Frh is responsible for most hydrogen production during growth of M. barkeri Fusaro on methanol, although some hydrogen is still produced in the Δ*frh* mutant. As will be shown below, Ech is probably responsible for the low levels of H_2_ seen in the Δ*frh* mutant.

10.1128/mBio.01256-18.2 TABLE S1  Methanosarcina barkeri Fusaro strains used in this study. Download TABLE S1, DOC file, 0.04 MB. Copyright © 2018 Kulkarni et al. 2018 Kulkarni et al. This content is distributed under the terms of the Creative Commons Attribution 4.0 International license.

### Vht activity is required for viability of M. barkeri.

To investigate the role of Vht during growth of M. barkeri, we attempted to delete the *vhtGACD* operon via homologous gene replacement (31, 32). However, despite numerous attempts, including selection on a variety of media, with and without supplementation of potential biosynthetic intermediates, no mutant colonies were obtained. We also attempted to delete the *vht* operon using the markerless deletion method of genetic exchange (33). This method relies on construction of a merodiploid strain with both mutant and wild-type alleles. Upon segregation of the merodiploid, 50% of the recombinants are expected to be mutants if there is no selective pressure against the mutant allele. However, if the mutation causes a reduction in growth rate (with lethality being the most extreme case), the probability of obtaining recombinants with the mutant allele is severely reduced. We tested 101 haploid recombinants obtained from a *vhtGACD*^*+*^/Δ*vhtGACD* merodiploid; all carried the wild-type *vht* allele. Taken together, these data suggest that the *vhtGACD* operon is critical for normal growth of M. barkeri.

**FIG 3:**
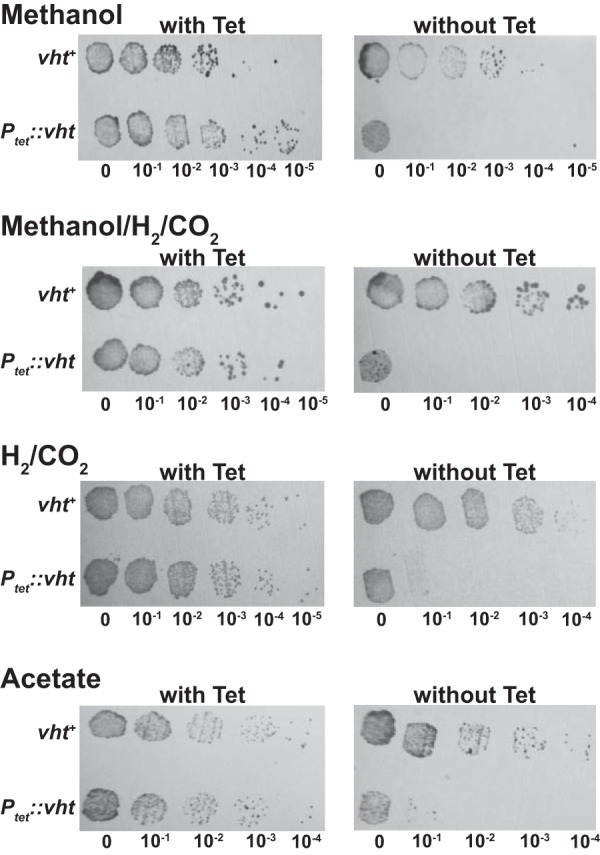
Essentiality of the Vht hydrogenase in M. barkeri. Cultures of the P_*tet*_::*vht* mutant (WWM157) and its isogenic parent (WWM154) were adapted to four different substrates of interest (and in the presence of tetracycline for P_*tet*_::*vht*), then washed, serially diluted, and incubated with each substrate with and without tetracycline (Tet). The media used indicate the ability to grow via each of the four known methanogenic pathways: (i) methylotrophic (methanol), (ii) methyl reduction (methanol/H_2_/CO_2_), (iii) hydrogenotrophic (H_2_/CO_2_), and (iv) aceticlastic (acetate).

**FIG 4:**
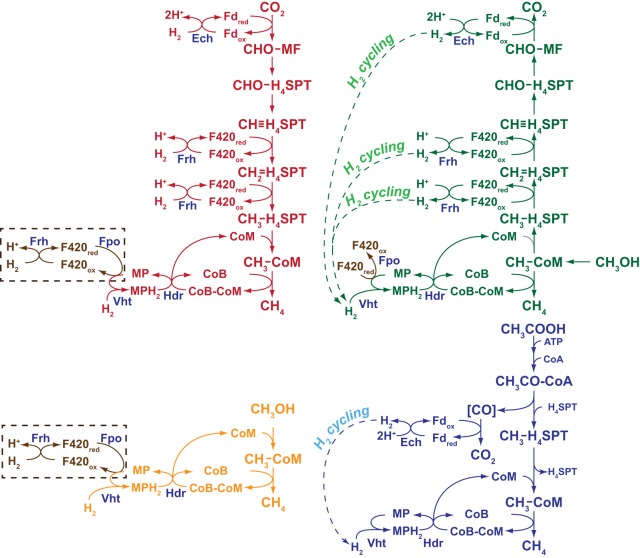
Role of H_2_ cycling in the four methanogenic pathways of M. barkeri. M. barkeri utilizes four distinct methanogenic pathways to allow growth on a variety of substrates. In the hydrogenotrophic pathway (shown in red), CO_2_ is reduced to methane using electrons derived from H_2_, while in the methyl-reducing pathway (shown in orange), H_2_ is used to reduce C_1_ compounds, such as methanol, directly to CH_4_. During methylotrophic methanogenesis (shown in green), C_1_ compounds are disproportionated to CO_2_ and methane, with one molecule of the C_1_ compound oxidized to provide electrons for reduction of three additional molecules to methane. Finally, in the aceticlastic pathway (shown in blue), acetate is split into a methyl group and an enzyme-bound carbonyl moiety. The latter is oxidized to CO_2_ to provide electrons required for reduction of the methyl group to methane. The steps catalyzed by Fpo, Frh, Vht, Ech, and Hdr proteins are indicated. Steps involving H_2_ cycling are shown by labeled dashed arrows. An alternate, H_2_-independent electron transport pathway is shown in brown. Experimental data support the function of this alternate pathway during methylotrophic methanogenesis, but not in hydrogenotrophic or methyl-reducing methanogenesis (as indicated by the dashed brown box). Abbreviations; Fpo, F420 dehydrogenase; Frh, F420-reducing hydrogenase; Vht, methanophenazine-dependent hydrogenase; Ech, energy-converting ferredoxin-dependent hydrogenase; Hdr, heterodisulfide reductase; CoM, coenzyme M; CoB, coenzyme B; CoB-CoM, mixed disulfide of CoB and CoM; MP/MPH_2_, oxidized and reduced methanophenazine; F420_ox_/F420_red_, oxidized and reduced cofactor F420, respectively; Fd_ox_/Fd_red_, oxidized and reduced ferredoxin, respectively; CHO-MF, formyl-methanofuran; H_4_SPT, tetrahydrosarcinapterin; CHO-H_4_SPT, formyl-H_4_SPT; CH≡H_4_SPT, methenyl-H_4_SPT; CH_2_=H_4_SPT, methylene-H_4_SPT; CH_3_-H_4_SPT, methyl-H_4_SPT; CH_3_-CoM, methyl-coenzyme M; CoA, coenzyme A; CH_3_CO-CoA, acetyl-coenzyme A; [CO], enzyme-bound carbonyl moiety.

To test whether Vht is essential, we constructed a mutant in which the *vht* operon was placed under control of a tightly regulated, tetracycline-dependent promoter (34). We then examined the viability of the mutant and its isogenic parent by spotting serial dilutions on a variety of media, with and without tetracycline. As shown in Fig. 3, the P_*tet*_::*vht* mutant is unable to grow in the absence of the inducer but grew well when tetracycline was added, whereas the isogenic parent grew with or without the addition of tetracycline. These phenotypes were observed on a variety of media, including media containing (i) methanol, (ii) methanol plus H_2_, (iii) H_2_/CO_2_, and (iv) acetate, which were chosen because they encompass growth conditions that require each of the four known methanogenic pathways used by M. barkeri (Fig. 4). It should be stressed that the P_*tet*_::*vht* mutant used in this experiment was pregrown in the presence of inducer. Thus, at the start of the experiment, all cells have active Vht. However, during cultivation in the absence of tetracycline, preexisting Vht is depleted by protein turnover and cell division, thereby allowing characterization of the Vht-deficient phenotype. The absence of growth of the diluted cultures in all media shows that Vht is essential for growth via the methylotrophic (methanol), methyl-reducing (methanol plus H_2_), hydrogenotrophic (H_2_/CO_2_), and aceticlastic (acetate) methanogenic pathways.

### Depletion of Vht results in H_2_ accumulation and cell lysis.

To help understand why Vht is essential, we quantified production of H_2_ and CH_4_ in cultures of the P_*tet*_::*vht* strain with and without tetracycline (Fig. 2). When the strains were grown in methanol-containing medium in the presence of tetracycline, the accumulation of H_2_ and CH_4_ was essentially identical to that of the isogenic parent. Cultures in which *vht* is not expressed (i.e., without tetracycline) grew initially but growth rapidly slowed and reached an optical density that was less than half of that obtained when *vht* was expressed. The optical density subsequently dropped, suggesting cell death and lysis. Similarly, methane accumulation in cultures not expressing *vht* was much slower than in induced cultures and only reached half of that seen under inducing conditions. In contrast, H_2_ accumulation was much higher in the absence of Vht, with final levels nearly sixfold higher than those seen in cultures that express Vht. These data clearly show that Vht is required for efficient recapture of H_2_ produced by Frh and Ech. Moreover, they suggest that H_2_ loss is responsible for the lethal consequences of *vht* repression.

### Vht is not essential in Δ*frh* mutants.

If the inability to recapture H_2_ is responsible for the essentiality of Vht, then it should be possible to delete the *vht* operon in strains that do not produce hydrogen. As described above, Frh is responsible for the majority of H_2_ production during growth. Thus, we attempted to introduce a Δ*vht* allele into the Δ*frh* host. In contrast to our prior unsuccessful attempts to create a Δ*vht* single mutant, the Δ*vht* Δ*frh* double mutant was isolated in the first attempt. Therefore, Vht is not required when Frh is absent. Like the Δ*frh* single mutant, the Δ*vht* Δ*frh* double mutant grows slowly on methanol and produces lower levels of methane (Fig. 2). Significantly, the double mutant does not produce the excessive level of H_2_ seen in the uninduced P_*tet*_::*vht* strain, instead accumulating H_2_ at levels similar to those of the parental strain (ca. 20 Pa). Because Ech is the only active hydrogenase remaining in the Δ*vht* Δ*frh* double mutant, it must be responsible for H_2_ production in this strain. This begs the question of why H_2_ accumulation stops at 20 Pa in the double mutant, while the uninduced P_*tet*_::*vht* strain produces much higher levels. We suggest that the coupling of Ech activity to generation of proton motive force thermodynamically restrains excessive H_2_ production, even in the absence of H_2_ uptake by Vht. This would also explain the viability of the Δ*vht* Δ*frh* double mutant. This situation is in stark contrast to that seen in the *vht*-depleted strain, where the F420-dependent Frh is responsible for most of the H_2_ production (see above). Accordingly, at the low H_2_ partial pressures observed in our experiments, reduction of protons with F420 is strongly exergonic, allowing excessive hydrogen accumulation. This is also consistent with the observation that the redox state of F420 is in rapid equilibrium with H_2_ (35). Interestingly, the smaller amount of H_2_ accumulation in the Δ*frh* mutant relative to that seen in the Δ*vht* Δ*frh* double mutant shows that Vht also consumes H_2_ produced by Ech. This supports previous studies indicating potential energy conservation via Ech/Vht H_2_ cycling during acetate metabolism (17, 23).

### M. barkeri has a bifurcated electron transport chain with H_2_-dependent and -independent branches.

We previously showed that M. barkeri has a branched electron transport chain, with Frh- and F420 dehydrogenase (Fpo)-dependent branches (16). The data reported here extend our understanding of the Frh-dependent branch and are fully consistent with the model depicted in Fig. 1. Thus, during growth on methylotrophic substrates such as methanol, reduced F420 is preferentially oxidized via an energy-conserving, H_2_ cycling electron transport chain that requires Frh. However, in the absence of Frh, reduced F420 is channeled into the Fpo-dependent electron transport chain, which supports growth at a significantly lower rate (Fig. 1 and 2). This alternate pathway accounts for the viability of the Δ*frh* mutant, which is lost when both *frh* and *fpo* are deleted (16). Similar but less severe phenotypes have been observed in *fpo* and *frh* mutants of Methanosarcina mazei, thus it seems likely that H_2_ cycling also occurs in this closely related species (36). However, many *Methanosarcina* species, especially those that inhabit marine environments, are devoid of hydrogenase activity, despite the presence of hydrogenase-encoding genes. We, and others, have interpreted this to be an adaptation to the marine environment, where H_2_-utilizing sulfate reducers are likely to disrupt H_2_ cycling due to the superior thermodynamics of H_2_ oxidation coupled to sulfate reduction (19, 37).

A similar branched electron transport chain may also explain the contradictory evidence regarding H_2_ cycling in *Desulfovibrio* species. Thus, the viability of *Desulfovibrio* hydrogenase mutants and the inability of excess H_2_ to suppress substrate catabolism can both be explained by the presence of alternative electron transport mechanisms. Indeed, metabolic modeling of Desulfovibrio vulgaris strongly supports this interpretation (13). Thus, it is critical that experiments designed to test the H_2_ cycling mechanism be interpreted within a framework that includes the possibility of branched electron transport chains. With this in mind, it seems likely that many anaerobic organisms might use H_2_ cycling for energy conservation. Indeed, since it was originally proposed, H_2_ cycling has been suggested to occur in the acetogen Acetobacterium woodii (10) and in the Fe(III) respiring Geobacter sulfurreducens (8).

### Why are Vht mutants inviable during growth on methanol/H_2_ or H_2_/CO_2_? 

Although the data presented here strongly support the H_2_ cycling model, they raise additional questions regarding H_2_-dependent methanogenesis that are not easily explained. In particular, it is not readily apparent why the uninduced P_*tet*_::*vht* mutants are inviable during hydrogenotrophic or methyl-reducing growth. As shown in Fig. 4, it should be possible to channel electrons from H_2_ oxidation into the electron transport chain via Frh and Fpo. Indeed, Thauer et al. have proposed that this alternate pathway is functional in *Methanosarcina* (38). Nevertheless, the P_*tet*_::*vht* mutant does not grow under repressing conditions on either H_2_/CO_2_ or methanol plus H_2_. It should be stressed that we use high concentrations of hydrogen during growth on these substrates. Thus, it is expected that reduction of F420 via Frh should be exergonic in our experiments, which would favor this pathway. (This is in contrast to the methylotrophic or aceticlastic growth conditions described above, under which the reverse reaction [i.e., hydrogen production] is favored.) Thus, a thermodynamic argument cannot easily explain the results. Further, based on available evidence (16, 39, 40), energy conservation via the Vht-dependent pathway should be identical to that of the alternate Frh/Fpo-dependent pathway. Thus, an energy conservation argument also cannot explain the phenomenon. One might argue that faster kinetics of the Vht-dependent pathway could be responsible, but in our opinion, the growth (albeit slower than wild type) of the Δ*frh* and Δ*vht* Δ*frh* mutants during methylotrophic growth, which depends on Fpo, argues against this explanation. Therefore, as yet unknown regulatory and/or biochemical constraints on hydrogen metabolism in *Methanosarcina* await discovery.

## MATERIALS AND METHODS

### Strains, media, and growth conditions.

The construction and genotypes of all *Methanosarcina* strains are presented in Table S1 in the supplemental material. *Methanosarcina* strains were grown as single cells (41) at 37°C in high-salt (HS) broth medium (42) or on agar-solidified medium as described previously (43). Growth substrates provided were methanol (125 mM in broth medium and 50 mM in agar-solidified medium) or sodium acetate (120 mM) under a headspace of either N_2_/CO_2_ (80/20%) at 50 kPa over ambient pressure or H_2_/CO_2_ (80/20%) at 300 kPa over ambient pressure. Cultures were supplemented as indicated with 0.1% yeast extract, 0.1% Casamino Acids, 10 mM sodium acetate, or 10 mM pyruvate. Puromycin (CalBioChem, San Diego, CA) was added at 2 µg/ml for selection of the puromycin transacetylase (*pac*) gene (33). 8-Aza-2,6-diaminopurine (8-ADP) (Sigma, St. Louis, MO) was added at 20 µg/ml for selection against the presence of *hpt* (33). Tetracycline was added at 100 µg/ml to induce the tetracycline-regulated P*mcrB*(*tet*O3) promoter (34). Standard conditions were used for growth of Escherichia coli strains (44) DH5α/λ-*pir* (45) and DH10B (Stratagene, La Jolla, CA), which were used as hosts for plasmid constructions.

### DNA methods and plasmid construction.

Standard methods were used for plasmid DNA isolation and manipulation using E. coli hosts (46). Liposome-mediated transformation was used for *Methanosarcina* as described previously (47). Genomic DNA isolation and DNA hybridization were performed as described previously (32, 42, 43). DNA sequences were determined from double-stranded templates by the W. M. Keck Center for Comparative and Functional Genomics, University of Illinois. Plasmid constructions are described in the supporting information (Tables S2 and S3).

10.1128/mBio.01256-18.3 TABLE S2  Plasmids used in this study. Download TABLE S2, DOC file, 0.05 MB. Copyright © 2018 Kulkarni et al. 2018 Kulkarni et al. This content is distributed under the terms of the Creative Commons Attribution 4.0 International license.

10.1128/mBio.01256-18.4 TABLE S3  Primers used in this study. Download TABLE S3, DOC file, 0.03 MB. Copyright © 2018 Kulkarni et al. 2018 Kulkarni et al. This content is distributed under the terms of the Creative Commons Attribution 4.0 International license.

### Construction of the Δ*frh* and Δ*vht* Δ*frh* mutants.

The markerless genetic exchange method (33) using plasmid pGK4 was employed to delete *frhADGB* (Δ*frh*) in the Δ*hpt* background of M. barkeri Fusaro (Tables S1, S2, and S3) using methanol/H_2_/CO_2_ as the growth substrate. The Δ*vht* Δ*frh* mutant was constructed by deleting *vhtGACD* in the Δ*frh* markerless mutant by the homologous recombination-mediated gene replacement method (32). To do this, the 5.6-kb XhoI/NotI fragment of pGK82B was used to transform the Δ*frh* mutant to puromycin resistance on methanol-containing medium. The mutants were confirmed by PCR and DNA hybridization (data not shown).

### Construction of the tetracycline-regulated *vht* mutant (P_*tet*_::*vht*).

The tetracycline-regulated P*mcrB*(*tet*O3) promoter was employed to drive conditional expression of the *vht* operon in M. barkeri WWM157 (34). This strain was constructed by transforming strain WWM154 to puromycin resistance using the 7-kb NcoI/SpeI fragment of pGK61A (Tables S1, S2, and S3). The transformants were selected on methanol plus H_2_/CO_2_ medium in the presence of puromycin and tetracycline. The P_*tet*_::*vht* strain was confirmed by DNA hybridization (data not shown). To ensure that the native *vht* promoter (P*vht*) did not interfere with expression from P*mcrB*(*tet*O3), 382 bp upstream of *vhtG* were deleted in P_*tet*_::*vht*. This left 1,038 bp intact for the expression of the *hyp* operon, which is upstream of the *vht* operon and expressed in the opposite direction.

### Determination of Vht essentiality during growth on all substrate types.

Growth of strains WWM157 (P_*tet*_::*vht*) and WWM154 (isogenic parent) on methanol, methanol/H_2_/CO_2_, H_2_/CO_2_, and acetate were analyzed by the spot-plate method (48). Cultures were first adapted for at least 15 generations to the substrate of interest; tetracycline was added to each medium for growth of strain WWM157. Upon reaching stationary phase, 10 ml of culture was washed three times and resuspended in 5 ml HS medium that lacked growth substrate. Subsequently, 10 µl of 10-fold serial dilutions was spotted onto the following: three layers of GB004 paper (Whatman, NJ), two layers of GB002 paper (Schleicher & Schuell BioScience, NH), one layer of 3 MM paper (Whatman, NJ), and a 0.22 mM nylon membrane (GE Water and Process Technologies, PA) soaked in 43 ml of HS medium containing the substrate of interest with and without tetracycline. The plates were sealed and incubated at 37°C for at least 2 weeks in an intrachamber anoxic incubator (49). Growth on acetate and methanol was tested under an atmosphere of N_2_/CO_2_/H_2_S (80/19.9/0.1 ratio), while growth on methanol/H_2_/CO_2_ or H_2_/CO_2_ was tested under an atmosphere of H_2_/CO_2_/H_2_S (80/19.9/0.1 ratio).

### Measurement of H_2_, CH_4_, and OD_600_ during growth on methanol.

M. barkeri WWM85 (isogenic parent), WWM157 (P_*tet*_::*vht*; grown in the presence of tetracycline), WWM115 (Δ*frh*), and WWM351 (Δ*vht* Δ*frh*) were grown on methanol until mid-exponential phase (optical density at 600 nm [OD_600_] of ca. 0.5) and then 1 ml (WWM85 and WWM157) or 5 ml (WWM115 and WWM351) was inoculated into 100 ml HS-methanol in a 500-ml serum bottle. For WWM157, the culture was washed once prior to inoculation with or without tetracycline. To measure H_2_ and CH_4_, ca. 1-ml or 2-ml headspace sample was withdrawn aseptically from the culture at various time points with a syringe that had been flushed with sterile, anaerobic N_2_. The gas sample was then diluted into 70 ml helium. A gas-tight syringe flushed with helium was subsequently used to withdraw 3 ml of the diluted sample, which was then injected into an SRI gas chromatograph, equipped with a reduction gas detector (RGD) and a thermal conductivity detector (TCD) at 52°C. The RGD column was a three-foot-long 13× molecule sieve, whereas the TCD column was a six-foot HayeSep D porous polymer column. The RGD column was used to detect H_2_ by peak height, and the TCD column was used to detect CH_4_ by peak area. Helium was used as the carrier gas. OD_600_ was also measured during the growth curve.
