# Supplementary figures and images for: Energy Conservation via Hydrogen Cycling in the Methanogenic Archaeon Methanosarcina barkeri
Source: mBio. 2018 Jul 3;9(4):e01256-18. doi: 10.1128/mBio.01256-18 (PMC6030560; doi:10.1128/mBio.01256-18)

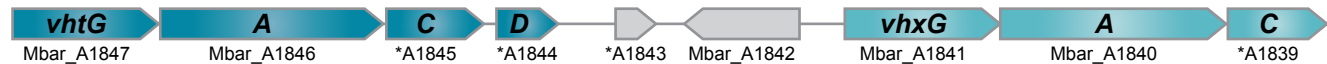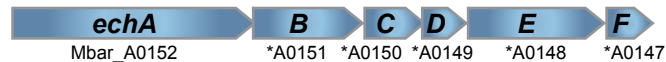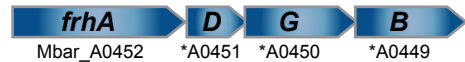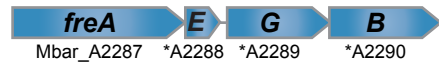

Supplement: FIG S1 [file mbo004183962sf1.pdf]
