# Supplementary material for: Energy Conservation via Hydrogen Cycling in the Methanogenic Archaeon Methanosarcina barkeri
Source: mBio. 2018 Jul 3;9(4):e01256-18. doi: 10.1128/mBio.01256-18 (PMC6030560; doi:10.1128/mBio.01256-18)
Supplement: TABLE S1 [file mbo004183962st1.doc]

**Table S1. *Methanosarcina barkeri* Fusaro strains used in this study**

| **Strain** | **Genotype** | **Source / Construction** |
| --- | --- | --- |
| WWM85 | ∆*hpt*::P*mcrB*-φC31*-int*-attP | (1) |
| WWM86 | ∆*hpt*::P*mcrB*-φC31*-int*-attB | (1) |
| WWM115 | ∆*frhADGB,*  ∆*hpt*::P*mcrB*-φC31*-int*-attB | Deletion of *frhADGB* by markerless exchange in WWM86 with pGK4. |
| WWM146 | ∆*vhtGACD*::*pac-hpt*, ∆*frhADGB,*  ∆*hpt*::P*mcrB*-φC31*-int*-attB | Deletion of *vhtGACD* by homologous gene replacement in WWM115 with XhoI/NotI-digested pGK82B. |
| WWM154 | ∆*hpt*::P*mcrB*-*tetR*-φC31*-int*-attB | (1) |
| WWM157 | P*mcrB*(tetO3)::*vhtGACD,* P*mcrB*-*tetR*-*pac*-*hpt,*^a^  ∆*hpt*::P*mcrB*-*tetR*-φC31*-int*-attB | Exchange of *vhtGACD* native promoter with Tc-regulated promoter in WWM154 with NcoI/SpeI-digested pGK61A. |
| WWM351 | ∆*vhtGACD*::FRT, ∆*frhADGB,*  ∆*hpt*::P*mcrB*-φC31*-int*-attB | Removal of *pac-hpt* from WWM146 by pMR55 mediated Flp recombination. |

^a^ The promoter replacement cassette from pGK61A (Table S2) includes a copy of *tetR* under control of the strong P*mcrB* promoter (P*mcrB*-*tetR*-*pac*-*hpt)* to ensure tight repression of the P*mcrB*(*tet*O3) promoter in the absence of tetracycline.

**References**

1. Guss AM, Rother M, Zhang JK, Kulkarni G, Metcalf W**W**. 2008. New methods for tightly regulated gene expression and highly efficient chromosomal integration of cloned genes fo*r Methanosarci*na species. Archaea 2:193-203.
