# Supplementary material for: Energy Conservation via Hydrogen Cycling in the Methanogenic Archaeon Methanosarcina barkeri
Source: mBio. 2018 Jul 3;9(4):e01256-18. doi: 10.1128/mBio.01256-18 (PMC6030560; doi:10.1128/mBio.01256-18)
Supplement: TABLE S2 [file mbo004183962st2.doc]

# Table S2. Plasmids used in this study.

| **Plasmid** | **Description and/or construction** | **Reference** |
| --- | --- | --- |
| pGK4 | AscI/KpnI-digested *frhADGB* upstream and downstream fusion PCR product amplified using primers frhupfor, frhuprev, frhdnfor and frhdnrev and ligated to MluI/KpnI-digested pMP44. | This study |
| pGK5 | AscI/NotI-digested *vhtGACD* upstream and downstream fusion PCR product amplified using primers vhtupfor, vhtuprev, vhtdnfor and vhtdnrev and ligated to MluI/NotI-digested pMP44. | This study |
| pGK51A | Vector containing a *pac*-*hpt* cassette and *tetR*; used to express genes from a tetracycline-regulated promoter (P*mcrB*(tetO3)) in *M*. *barkeri* Fusaro. | (1) |
| pGK55 | ApaI/NcoI-digested *vhtGACD* upstream region amplified using primers Tcvhtupfor and Tcvhtuprev and ligated to ApaI/NcoI-digested pGK51A. | This study |
| pGK61A | NdeI/SpeI-digested *vhtGACD* coding region amplified using primers Tcvhtcodfor and Tcvhtcodrev and ligated to NdeI/SpeI-digested pGK55. | This study |
| pGK82A | XhoI/HindIII-digested *vhtGACD* upstream region amplified using primers vhtdoubleupfor and vhtdoubleuprev and ligated to XhoI/HindIII-digested pJK301. | This study |

**Table S2 continued. Plasmids used in this study.**

| **Plasmid** | **Description and/or construction** | **Reference** |
| --- | --- | --- |
| pGK82B | SpeI/NotI-digested *vhtGACD* downstream region amplified using primers vhtdoublednfor and vhtdoublednrev and ligated to SpeI/NotI-digested pGK82A. | This study |
| pJK301 | Vector containing a *pac*-*hpt* cassette; used to delete genes from *M*. *barkeri* Fusaro chromosome using double homologous recombination-mediated gene replacement. | (2) |
| pMP44 | Vector containing a *pac*-*hpt* cassette; used to delete genes from *M*. *barkeri* Fusaro chromosome using the markerless exchange method. | (3) |
| pMR55 | Non-replicating plasmid that contains the Flp recombinase gene under control of P*mcrB.* | (4) |

**References**

1. Guss AM, Rother M, Zhang JK, Kulkarni G, Metcalf WW. 2008. New methods for tightly regulated gene expression and highly efficient chromosomal integration of cloned genes for *Methanosarcina* species. Archaea 2:193-203.

2. Welander PV, Metcalf WW. 2008. Mutagenesis of the C1 oxidation pathway in *Methanosarcina barkeri*: new insights into the Mtr/Mer bypass pathway. J Bacteriol 190:1928-1936.

3. Pritchett MA, Zhang JK, Metcalf WW. 2004. Development of a markerless genetic exchange method for *Methanosarcina acetivorans* C2A and its use in construction of new genetic tools for methanogenic archaea. Appl Environ Microbiol 70:1425-1433.

4. Rother M, Metcalf WW. 2005. Genetic technologies for Archaea. Curr Opin Microbiol 8:745-51.
