# Supplementary material for: Energy Conservation via Hydrogen Cycling in the Methanogenic Archaeon Methanosarcina barkeri
Source: mBio. 2018 Jul 3;9(4):e01256-18. doi: 10.1128/mBio.01256-18 (PMC6030560; doi:10.1128/mBio.01256-18)
Supplement: TABLE S3 [file mbo004183962st3.doc]

# Table S3. Primers used in this study

| **Primer** | **Sequence^a^** |
| --- | --- |
| frhupfor | GGCGCGCCTCCGTTGTCCTTCTTTCCAC |
| frhuprev | CATCCCCGTATTCAGCGTAGAGGCTGTACCGTGGTTAAGG |
| frhdnfor | CCTTAACCACGGTACAGCCTCTACGCTGAATACGGGGATG |
| frhdnrev | GGCGCGCCGGTACCTGTTGCGAGTTGTTCAATCC |
| Tcvhtupfor | GGCGCGCCCCATGGGAAGTTTTCGGGGGTCTTTC |
| Tcvhtuprev | GGCGCGCCGGGCCCTGCATGAAAAGAAAATAAAATGC |
| Tcvhtcodfor | GGCGCGCCCATATGAGTACTGGAATAAAAAATCTTGTC |
| Tcvhtcodrev | GGCGCGCCACTAGTCCTGCGTCTTTGGAGAAATC |
| vhtupfor | GGCGCGCCAAGGTGAATTCCCGTTTTCC |
| vhtuprev | GTGAAGAAAATAATTGAACAAAACAAATCTTGATTTTACCCAAATTATACATACG |
| vhtdnfor | CGTATGTATAATTTGGGTAAAATCAAGATTTGTTTTGTTCAATTATTTTCTTCAC |
| vhtdnrev | GGCGCGCCGCGGCCGCTTTGGACTTTCCCGTACCTG |
| vhtdoubleupfor | GGCGCGCCCTCGAGAAGGTGAATTCCCGTTTTCC |
| vhtdoubleuprev | GGCGCGCCAAGCTTTCTTGATTTTACCCAAATTATACATACG |
| vhtdoublednfor | GGCGCGCCACTAGTTTTGTTTTGTTCAATTATTTTCTTCAC |
| vhtdoublednrev | GGCGCGCCGCGGCCGCTTTGGACTTTCCCGTACCTG |

^a^ Introduced restriction endonuclease recognition sequences are single underlined. Double underline indicates introduced overlap region for fusion PCR.
